# UPP1 in Cancer: Context-Dependent Roles in Metabolic Adaptation and Treatment Response

**DOI:** 10.3390/cimb48080813

**Published:** 2026-08-12

**Authors:** Tong Cai, Xuyu Chen, Juanjuan Huang, Yukun Xu, Juan Li

**Affiliations:** 1 Cancer Medical Center, The Second Affiliated Hospital of Nanjing Medical University, Nanjing 210000, China; caitongstudent@stu.njmu.edu.cn (T.C.); huangjuanjuan223@163.com (J.H.); 18167277067@163.com (Y.X.); 2 Department of Gastroenterology, The Affiliated Hospital of Yangzhou University, Yangzhou 225001, China; chenxuyu@stu.njmu.edu.cn

**Keywords:** uridine phosphorylase 1, uridine catabolism, pyrimidine salvage, cancer metabolism, tumor microenvironment, fluoropyrimidine response

## Abstract

Uridine phosphorylase 1 (UPP1) catalyzes the reversible phosphorolysis of uridine to uracil and ribose-1-phosphate, thereby linking pyrimidine salvage to central carbon metabolism. In cancer, UPP1 is functionally relevant when UPP1-linked pathway engagement and a perturbation-dependent phenotype converge within a defined cellular, nutrient/substrate, molecular, or treatment setting. The direction of effect is determined by the dominant biochemical or signaling output, whereas the strength of UPP1 dependence may be attenuated by compensation through related nucleoside-metabolic pathways. This review examines the roles of UPP1 in metabolic adaptation under nutrient stress, remodeling of the tumor microenvironment, and treatment response across cancer types. The strongest evidence concerns uridine-derived ribose-carbon utilization under glucose limitation and modulation of fluoropyrimidine response by UPP1. Mechanistic support for immune and microenvironmental effects is strongest in lung adenocarcinoma and selected metabolite-centered models, whereas most patient-outcome and pan-cancer findings remain cohort- or signature-based. This heterogeneity argues against treating UPP1 as a universal oncogenic driver or a stand-alone pan-cancer biomarker. Its biological and translational value lies in identifying tumor states in which UPP1-linked metabolism becomes functionally important or therapeutically informative.

## 1. Introduction

Worldwide, cancer remains a leading cause of death [1]. Metabolic reprogramming is a defining feature of cancer and contributes to tumor progression and treatment response beyond energy supply alone [2]. Its effects extend to the tumor microenvironment (TME), where nutrient competition and metabolite accumulation can reshape immune states and influence immune evasion and therapeutic sensitivity [3,4,5,6,7]. Single-cell and spatial omics now permit these metabolism–immunity interactions to be examined at cellular resolution [8]. Specific metabolic enzymes can thereby connect metabolic state with microenvironmental remodeling and treatment response [9].

Pyrimidine metabolism is central to cancer biology because it directly affects nucleic acid synthesis and drug response [10,11,12]. Alongside de novo biosynthesis, salvage-associated branches contribute to metabolic adaptation under therapeutic pressure, with implications for fluoropyrimidine efficacy and, in selected clinical settings, nucleoside-analogue response [13,14]. Under nutrient limitation, nucleosides can also supply carbon to central metabolism and buffer tumor-cell stress [15,16].

UPP1 has been linked experimentally to functions beyond canonical salvage, including microenvironmental remodeling and metastasis-associated phenotypes [17]. The central question is which tumor states make UPP1 functionally important, what determines the direction of its effects, and whether related pathways compensate for its loss.

## 2. Biochemical Positioning of UPP1 in Uridine Catabolism

UPP1 mediates the reversible phosphorolysis of uridine, yielding uracil and ribose-1-phosphate (R1P) in the presence of inorganic phosphate [18,19,20]. By generating both a reusable pyrimidine base and a phosphorylated ribose intermediate, this reaction places UPP1 at the intersection of nucleoside turnover and central carbon metabolism. The molecular identity of the enzyme later designated UPP1 was established in the 1990s, when its human cDNA was cloned from HCT116 cells and the catalytic activity of the recombinant enzyme was confirmed [19]. Structural studies further show conformational plasticity in human UPP1 during catalysis, which coordinates substrate binding and product release [18].

Steady-state kinetic analysis of recombinant human UPP1 identified an ordered bi–bi mechanism in which inorganic phosphate binds before uridine and uracil is released before R1P [20]. These purified-enzyme measurements define the catalytic sequence; tumor-specific pathway flux under nutrient or treatment conditions was not measured.

Pyrimidine salvage is classically regarded as a less energy-intensive complement to de novo nucleotide synthesis [21,22]. Under nutrient limitation, UPP1-derived R1P can connect uridine catabolism to central carbon metabolism through the non-oxidative pentose phosphate pathway [16]. The model-specific tracer evidence and downstream glycolytic outputs are examined in Section 3.1. This pathway extends the functional significance of nucleoside catabolism beyond nucleotide provision [15].

Uridine homeostasis is governed by several interconnected pathways, including related nucleoside phosphorylases, kinase-mediated salvage, RNA uridylation, and systemic metabolic signaling [23,24,25,26,27,28,29]. Although UPP2 catalyzes the same reversible phosphorolysis of uridine as UPP1, it is predominantly expressed in the kidney and subject to redox-dependent inactivation—a feature not observed in UPP1 [23,28]. TYMP represents a structurally distinct phosphorylase with a marked preference for thymidine and other 2′-deoxyribosides [24], whereas UCK1 and UCK2 salvage uridine and cytidine via ATP-dependent phosphorylation to generate UMP and CMP rather than through phosphorolytic cleavage [25]. Consequently, the biological relevance of UPP1 stems not from exclusive catalytic specificity for uridine, but rather from its capacity to couple uridine availability and R1P production to carbon utilization and treatment response across defined tumor contexts [16,18,30,31,32,33,34,35].

## 3. Mechanistic Contexts of UPP1 in Tumors

**Figure 1:**
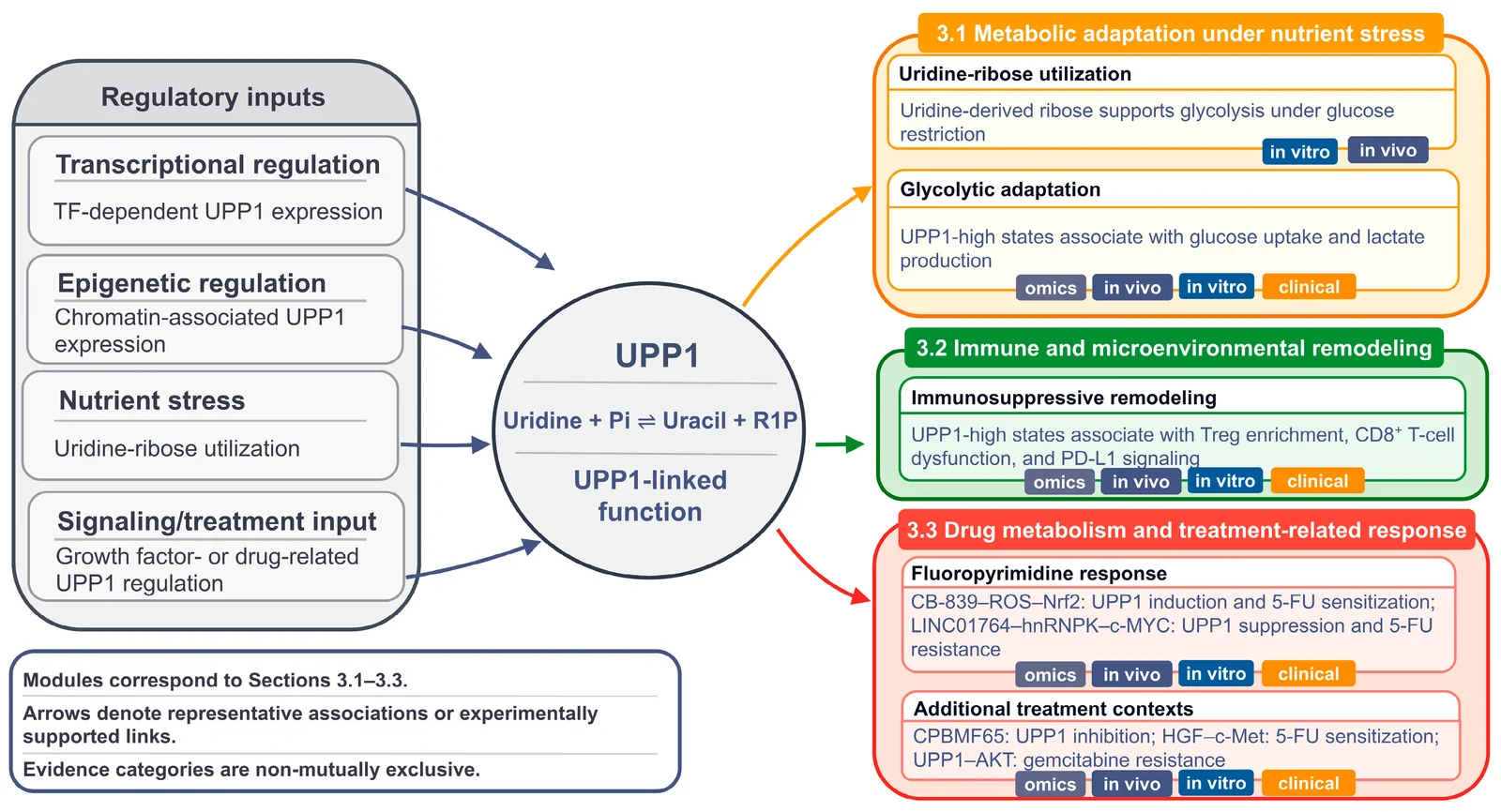
Framework Linking UPP1 to Three Recurrent Functional Settings. Schematic overview of transcriptional, epigenetic, metabolic, and signaling inputs associated with UPP1 and their relationships to metabolic adaptation under nutrient stress, immune and microenvironmental remodeling, and treatment-related response. Gray boxes indicate regulatory inputs, whereas orange, green, and red boxes identify the three functional settings: metabolic adaptation under nutrient stress, immune and microenvironmental remodeling, and drug metabolism and treatment-related response, respectively. Evidence labels indicate omics, in vitro, in vivo, or clinical support and are non-mutually exclusive. Clinical evidence includes patient-derived, retrospective, or exploratory data where applicable and does not imply prospective validation. For boxes containing multiple routes, evidence labels summarize the combined evidence across routes and do not apply to every listed route. Arrows denote reported associations or experimentally supported links, as specified by the accompanying evidence labels. The figure was created by the authors using Microsoft PowerPoint.

**Figure 2:**
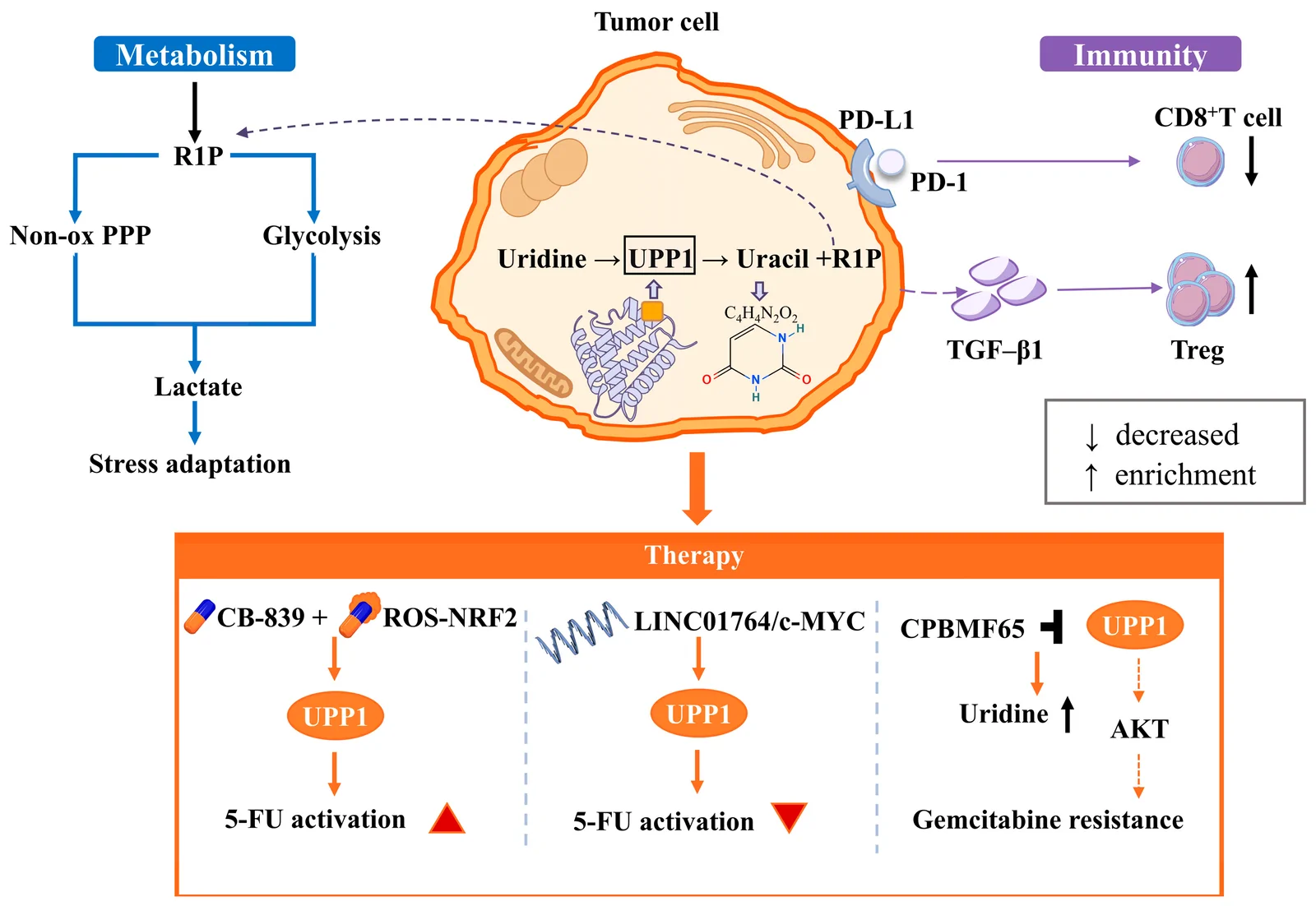
UPP1-Centered Uridine Links Metabolic Adaptation, Immune Remodeling, and Treatment-Related Response. UPP1-mediated uridine catabolism generates R1P, and the metabolic panel summarizes the experimentally traced redistribution of uridine-derived ribose carbon into central metabolism. In specific models, UPP1 has also been linked to immunosuppressive outputs and treatment-specific changes in chemotherapy response. Blue, purple, and orange color coding identifies the metabolic, immune, and treatment-related components, respectively. Arrows indicate the direction of the depicted biochemical flow or reported regulatory/phenotypic relationships. The therapy panel presents distinct model-supported routes related to fluoropyrimidine response, enzymatic inhibition, and gemcitabine resistance rather than a single linear downstream cascade. Abbreviations: 5-FU, 5-fluorouracil; AKT, protein kinase B; Nrf2, nuclear factor erythroid 2-related factor 2; PD-L1, programmed death-ligand 1; PPP, pentose phosphate pathway; R1P, ribose-1-phosphate; Treg, regulatory T cell. The figure was assembled by the authors using Microsoft PowerPoint. Protein illustration: Created in BioRender. Cai, T. (2026) https://BioRender.com/ckqoxt3, accessed on 25 June 2026. The receptor and RNA illustrations were adapted from Servier Medical Art. The cell illustration was adapted from “cell” by Alexander Bates, SciDraw (DOI: 10.5281/zenodo.4420077), and the capsule illustration was adapted from “Pill Capsule Medicine” by Daniel Clough, SciDraw (DOI: 10.5281/zenodo.3926307). These Servier Medical Art and SciDraw illustrations were used under the Creative Commons Attribution 4.0 International license. The uracil structure was obtained from PubChem (CID 1174), National Center for Biotechnology Information, U.S. National Library of Medicine.

UPP1-linked phenotypes vary across tumor and treatment models with nutrient state, cellular source, regulatory program, and therapeutic exposure. Pan-cancer analyses associate UPP1 expression with tumor-microenvironment programs involving immune-cell organization, cytokine and immune-checkpoint activity, and invasion-associated features [26], while perturbation-based models connect UPP1 to extracellular-matrix remodeling and metastatic progression [17]. Three recurrent mechanistic settings emerge: metabolic adaptation under nutrient stress, immune and microenvironmental remodeling, and drug metabolism and treatment-related response. Figure 1 maps the principal regulatory inputs and functional outputs across these settings; Figure 2 centers on the biochemical route from uridine phosphorolysis to ribose-carbon utilization.

Here, “context” denotes the combined nutrient and substrate environment, the cellular source and spatial localization of UPP1, the molecular programs governing its expression or downstream signaling, and treatment exposure. For the purposes of this review, we distinguish a functionally supported context from an association-only context using three criteria: (i) UPP1-linked pathway engagement, demonstrated by substrate turnover, isotope incorporation, drug-metabolite formation, or a target-proximal signaling readout; (ii) a reproducible phenotype after genetic or pharmacological perturbation of UPP1, with rescue or epistasis where available; and (iii) measurement of the pathway readout and phenotype within the same defined cellular source, nutrient/substrate condition, molecular background, tumor model, or treatment setting. When these elements do not converge, expression or outcome associations identify candidate settings for mechanistic testing rather than established UPP1 function. A stronger claim of a non-redundant metabolic dependency further requires evidence that related nucleoside-metabolic enzymes do not fully compensate for UPP1 loss, which has rarely been tested directly [23,24,28].

The direction of effect should be interpreted according to the dominant UPP1-linked output rather than inferred from UPP1 abundance alone. Uridine-derived carbon use can support survival and growth during nutrient restriction [16,32]; tumor-cell UPP1-linked cytokine and immune-checkpoint signaling can promote immunosuppressive remodeling in lung adenocarcinoma [36]; enhanced fluoropyrimidine activation can increase drug sensitivity, whereas restricted activation can favor resistance [33,34,35]; UPP1–AKT signaling can reduce DCK-dependent gemcitabine activation and promote resistance [37]; and UPP1 activity in stromal or immune cells can alter tumor behavior through extracellular uridine depletion or uracil-mediated niche remodeling [17,38]. Thus, similar changes in UPP1 abundance can produce different phenotypes when substrate availability, cellular source, molecular program, or treatment exposure differs.

**Figure 3:**
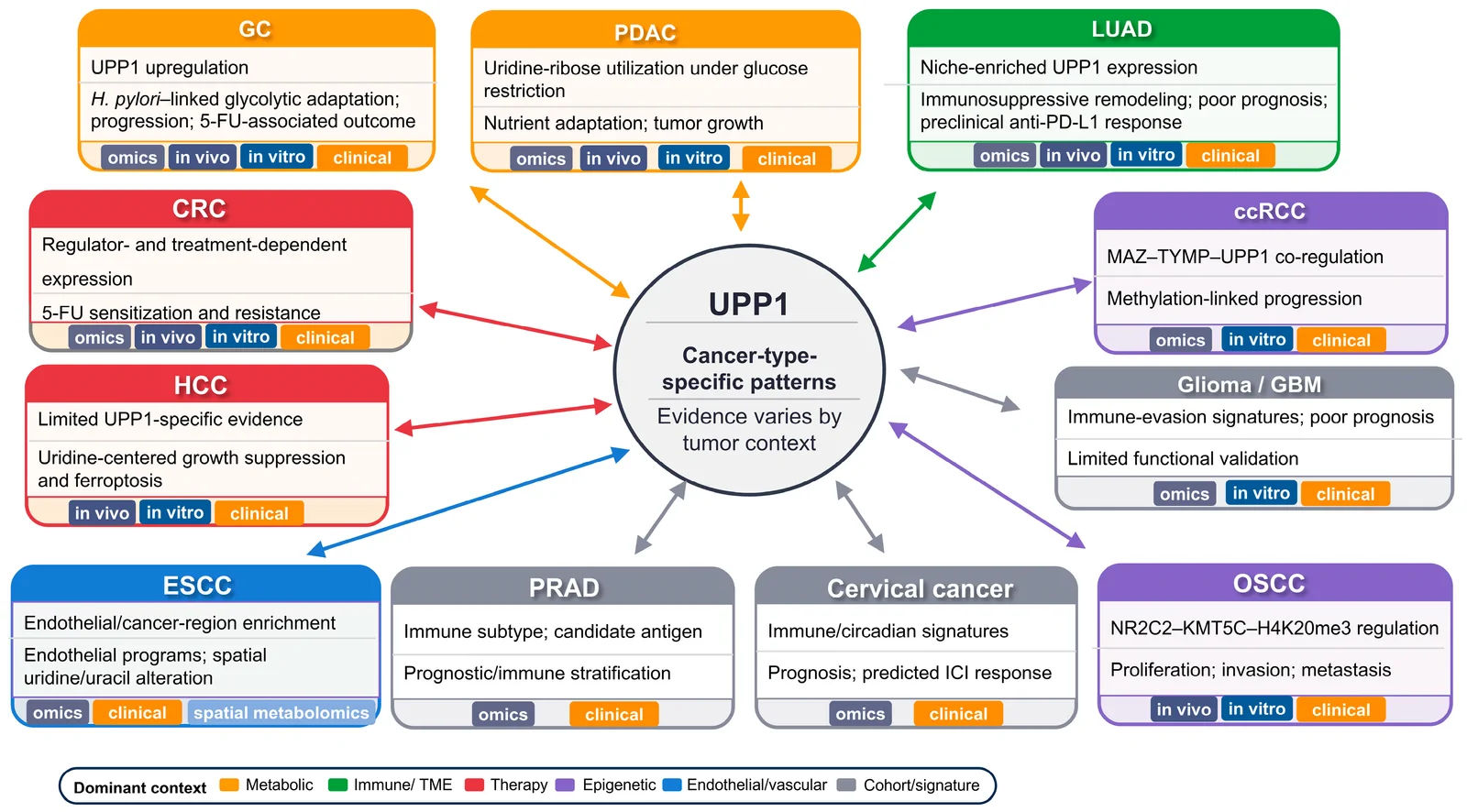
Selected Cancer-Type-Specific Overview of UPP1-Associated Patterns and Evidence Boundaries. The cards summarize selected UPP1 expression patterns, biological contexts, reported outcomes, and evidence bases across tumor types. Header colors indicate the dominant biological context. Evidence labels indicate omics, in vitro, in vivo, or clinical support and are non-mutually exclusive. Clinical evidence includes patient-derived, retrospective, or exploratory data where applicable and does not imply prospective validation. Within each card, evidence labels summarize the combined evidence for the listed pattern and outcomes and do not apply to every item individually. Abbreviations: CRC, colorectal cancer; ESCC, esophageal squamous cell carcinoma; GC, gastric cancer; GBM, glioblastoma; HCC, hepatocellular carcinoma; ICI, immune checkpoint inhibitor; LUAD, lung adenocarcinoma; OSCC, oral squamous cell carcinoma; PDAC, pancreatic ductal adenocarcinoma; PRAD, prostate adenocarcinoma; ccRCC, clear cell renal cell carcinoma. The figure was created by the authors using Microsoft PowerPoint.

Evidence is classified into four non-mutually exclusive categories: transcriptomic or other omics associations, in vitro functional evidence, in vivo evidence, and clinical evidence. Clinical evidence encompasses patient-derived observations and retrospective or exploratory analyses; the label does not denote prospective validation. These categories are applied throughout the tabulated summaries and in the evidence labels in Figure 1 and Figure 3.

Association-based observations are therefore distinguished from perturbation-supported effects and pathway-resolved mechanisms, and in vivo evidence is identified explicitly where available.

Representative mechanistic axes across these settings are summarized in Table 1. 

### 3.1. Metabolic Adaptation Under Nutrient Stress

Under nutrient limitation, uridine catabolism provides a route for ribose-carbon redistribution beyond nucleotide salvage. Choi et al. framed uridine as a metabolic “currency” that connects organismal uridine availability with multiple downstream metabolic fates [44]. Direct pathway evidence comes from ribose-labeled [^13^C_5_]uridine tracing. Calculated from the published source-data peak areas, M+5 represented approximately 91% of the total ribose-phosphate signal and M+3 approximately 86% of the total lactate signal in UPP1-FLAG K562 cells after 5 h with 5 mM [^13^C_5_]uridine, compared with approximately 16% and 3%, respectively, in GFP-control cells. Complementary gain- and loss-of-function experiments supported UPP1/UPP2 dependence of uridine use, and fasted mice showed lower but measurable labeling in liver ribose-phosphate, lactate, and glucose 30 min after tracer administration [16].

In glucose-restricted pancreatic ductal adenocarcinoma (PDAC) cells, uridine, UMP, and UTP were more than 90% M+5-labeled after 24 h with 1 mM [^13^C_5_]uridine. At 0.1 mM glucose plus 0.1 mM tracer, uridine-derived carbon exceeded 50% enrichment in some pentose-phosphate-pathway, glycolytic, and tricarboxylic-acid-cycle metabolites. In syngeneic tumors, nearly 30% of the tumor uridine pool was labeled 1 h after tracer administration, whereas UPP1 knockout restricted downstream labeling in glycolytic and tricarboxylic-acid-cycle intermediates in vitro [32]. Gastric cancer provides an inflammation-linked example: integrated omics, patient-tissue analyses, cell-based perturbation, and mouse gastric organoids connected *H. pylori*–NF-κB/p65-dependent UPP1 induction with uridine-supported energy production and growth under glucose restriction [41].

Regulatory backgrounds differ across these adaptive states. In colorectal cancer, the CBFβ–RUNX2 complex directly activates UPP1 transcription, although that study did not quantify uridine turnover or ribose-carbon utilization [40]. HOTAIR recruits EZH2 to the UPP1 promoter and alters H3K27me3-associated transcriptional regulation; cell-based perturbation and uridine-supported growth assays further linked this axis to UPP1-dependent metabolic phenotypes [42]. In gastric models, single-cell, patient-derived, and cell-based analyses independently associated *H. pylori*–NF-κB/p65 signaling with UPP1 activation [45], while the later organoid study connected this regulatory program to nutrient-restricted uridine utilization [41]. Across these models, transcriptional activation is established more broadly than downstream catabolic use, which was examined directly in the organoid study.

Functional metabolic evidence outside tracer-defined models comes from lung adenocarcinoma, where UPP1 upregulation increased glucose uptake, lactate production, and glycolytic activity and increased sensitivity to glycolytic inhibition in vitro and in vivo [39]. By contrast, UPP1-enriched fibroblasts in metastatic pancreatic cancer [46], pan-cancer tumor-microenvironment associations [26], and a UPP1-containing glioblastoma prognostic signature [47] identify candidate cellular or disease settings rather than UPP1-dependent carbon utilization. The tracer datasets quantify fractional isotopologue enrichment at defined time points rather than model-derived absolute flux, which would require additional temporal and mass-balance constraints [16,32,48,49]. Perturbation-coupled tracing therefore provides the most direct evidence for nutrient-stress adaptation, whereas single-cell and transcriptomic studies identify cellular sources and tumor settings for direct metabolic testing.

### 3.2. Immune and Microenvironmental Remodeling

Metabolic competition, hypoxic stress, and changes in nutrient and metabolite availability contribute to immune and stromal organization within tumors [50,51]. UPP1-related evidence spans four levels: single-cell, bulk-transcriptomic, and tissue analyses define associated cell states and spatial niches; perturbation, cytokine, co-culture, and in vivo studies test immune function or signaling; metabolite measurements identify candidate extracellular mediators; and substrate-competition models examine how UPP1 activity in non-malignant cells alters tumor nutrient access [17,36,38,52,53,54,55]. Together, these studies address three distinct processes: immunosuppressive cell-state shifts, checkpoint regulation, and metabolite- or niche-mediated remodeling.

#### 3.2.1. Immunosuppressive Cell-State Shifts

Nutrient scarcity and metabolite accumulation can constrain immune-cell metabolism and favor suppressive organization in the tumor microenvironment [50,51]. T-cell effector activity, in particular, depends on glycolytic support for cytokine and effector-molecule production [56], and glycolytic states can intersect with the PD-1/PD-L1 axis [57].

Lung adenocarcinoma provides the most developed tumor-cell-centered model. Single-cell and bulk transcriptomic analyses, supported by patient-tissue profiling, identified UPP1-high tumor cells within invasion-associated niches enriched for Tregs, exhausted CD8^+^ T cells, M2-like macrophages, and fibroblast-associated states. Tumor-cell perturbation, co-culture assays, and syngeneic models showed that UPP1 upregulation reduced CD8^+^ T-cell activity and promoted broader immunosuppressive remodeling [36].

Glioma and glioblastoma studies combine cohort associations with narrower cell-based assays. Multi-cohort analyses link UPP1 expression to immune-cell infiltration and immune-related programs [52]. In glioma cells, UPP1 silencing reduced tumor-cell proliferation and migration and reduced HMC3 microglial migration toward glioma cells in a co-culture Transwell assay [53]. A nucleotide-metabolism signature included UPP1 without isolating its contribution [54]. Functional testing currently covers tumor-cell behavior and tumor-cell–microglial migration, not the broader immune and checkpoint programs observed in cohorts [52,55].

#### 3.2.2. Immune Checkpoint Activation

Hypoxia can induce PD-L1 through HIF-1α and strengthen MDSC-mediated T-cell suppression, providing a general metabolic framework for the checkpoint phenotype observed in UPP1-high tumors [58].

In lung adenocarcinoma, UPP1 expression correlated with PD-L1 across single-cell, bulk, and patient-tissue analyses. Genetic perturbation and pathway-rescue experiments further placed PI3K–AKT–mTOR signaling between UPP1 and PD-L1 expression. UPP1 upregulation also increased TGF-β1 secretion and impaired CD8^+^ T-cell cytotoxicity, while UPP1 inhibition improved the antitumor effect of PD-L1 blockade in mouse models [36]. The perturbation, signaling-rescue, co-culture, and immunocompetent in vivo experiments demonstrate a UPP1-dependent checkpoint-regulatory mechanism in the tested LUAD models [36].

In IDH-wild-type glioblastoma, high UPP1 expression is associated with PD-1-, PD-L1-, CTLA-4-, and CD70-related patterns, whereas experiments assessed tumor-cell migration rather than checkpoint signaling or immune-cell function [55]. The broader literature on glycolysis and the PD-1/PD-L1 axis supports biological plausibility but provides no UPP1-specific test [57].

#### 3.2.3. Metabolite and Niche-Mediated Remodeling

A mammary-tumor metastasis model provides functional evidence that neutrophil-associated UPP1 activity contributes to pre-metastatic-niche remodeling. Untargeted and targeted LC–MS showed that circulating uracil was associated with lung metastatic burden, and isotope-labeled uridine assays in isolated neutrophils demonstrated UPP1-dependent uracil production [17]. UPP1-expressing neutrophils sup-pressed CD8^+^ T-cell proliferation, whereas uracil altered fibroblast α5β1-integrin trafficking and increased fibronectin deposition. Genetic loss or pharmacological inhibition of UPP1 increased pulmonary T-cell abundance, reduced fibronectin accumulation, and lowered lung metastatic incidence [17]. These experiments linked a measured UPP1 product to both immune suppression and extracellular-matrix remodeling. Cell-source attribution came from neutrophil assays and localization; the study did not include neutrophil-specific Upp1 deletion or niche-level uracil concentration and turnover measurements.

A distinct stromal model used CRISPRa-engineered adipocytes to increase UPP1 expression and compete with pancreatic cancer cells for uridine. Radiolabeled-uridine uptake, low-glucose co-culture, uridine rescue, and ATP, NADH, and lactate measurements demonstrated substrate competition and reduced tumor-cell metabolism in the engineered adipocyte model; UPP1-activated adipose organoids also reduced xenograft growth [38]. The study measured substrate competition and tumor-cell metabolism, without immune endpoints or direct quantification of intratumoral uridine depletion.

#### 3.2.4. Evidence Summary

Across these models, cellular source separates the mechanisms more clearly than UPP1 expression level. Tumor-cell UPP1 regulates cytokine and checkpoint signaling in lung adenocarcinoma, neutrophil UPP1 generates uracil linked to pre-metastatic-niche remodeling, and engineered adipocyte UPP1 limits tumor-cell uridine access [17,36,38]. These source-specific outputs are mechanistically distinct and should not be collapsed into a single UPP1-defined immunosuppressive state.

### 3.3. Drug Metabolism and Treatment-Related Response

UPP1 affects treatment response through two mechanistically distinct routes: fluoropyrimidine anabolism and signaling-mediated resistance to other nucleoside analogues. The pharmacological importance of uridine phosphorylase activity was recognized before isoform-specific attribution was possible. Early work showed that uridine phosphorylase converts 5′-deoxy-5-fluorouridine (5′-DFUR) to 5-FU: an L1210 subline lacking this activity was resistant to 5′-DFUR but remained sensitive to 5-FU, whereas sarcoma-180 tumors with abundant activity accumulated higher concentrations of locally generated 5-FU [59]. Studies in gastric and colorectal cancer models subsequently identified two routes of 5-FU anabolism: direct conversion to fluorouridine monophosphate by orotate phosphoribosyltransferase and an indirect sequence involving uridine phosphorylase followed by uridine kinase. The relative contribution of these routes varied among cell lines and xenografts [60]. Because these studies did not resolve uridine-phosphorylase isoforms, they establish a historical enzymatic framework rather than UPP1-specific evidence.

More recent studies define UPP1-dependent effects in selected molecular settings. In PIK3CA-mutant colorectal cancer, CB-839 increased oxidative stress and Nrf2 activity, induced UPP1, and facilitated 5-FU anabolism; UPP1 knockout attenuated the antitumor effect of CB-839 plus 5-FU in xenografts [33]. LINC01764 instead promotes hnRNPK-dependent c-MYC translation and glucose and glutamine metabolism while suppressing UPP1. Patient-cohort, cell-based, and mouse data linked the broader LINC01764 program to poor chemotherapy response and 5-FU resistance; the UPP1-specific connection to impaired 5-FU activation was established in vitro [34]. Thus, UPP1 induction can facilitate fluoropyrimidine activation and sensitization, while its suppression can restrict activation and favor resistance.

HepG2 studies illustrate two additional forms of treatment-related modulation. CPBMF65 directly inhibited UPP1, increased intracellular uridine, and reduced proliferation through cell-cycle arrest and senescence in vitro [43]. Conversely, HGF increased UPP1 expression and intracellular 5-fluorouridine formation, thereby strengthening 5-FU cytotoxicity [35].

A distinct mechanism operates in bladder cancer. UPP1 binds the AKT C terminus and facilitates AKT activation; genetic perturbation, AKT-directed rescue experiments, and xenograft studies demonstrated that this signaling function promotes malignant phenotypes and gemcitabine resistance in the tested bladder-cancer models [37]. Downstream, UPP1–AKT signaling suppresses the FOXO1–DCK axis. Because DCK phosphorylates gemcitabine to initiate its intracellular activation, reduced DCK expression lowers drug sensitivity. Opposite UPP1 states can therefore compromise different treatments: low UPP1 restricts fluoropyrimidine activation, whereas high UPP1 promotes AKT-dependent suppression of gemcitabine activation [34,37]. These studies measured intracellular 5-fluorouridine [35], fluoropyrimidine metabolites and nucleotide pools [33], and DCK-centered signaling [37], but not UPP1-specific catalytic kinetics or model-derived absolute drug-metabolite flux.

## 4. Cancer-Specific Patterns of UPP1

This section compares cancer types by UPP1 expression pattern, cellular source, molecular background, dominant biological context, and evidence depth. Figure 3 provides a cancer-card overview, with header colors denoting dominant contexts and evidence labels indicating the principal evidence categories.

Table 2 aligns UPP1 expression patterns, biological contexts, reported outcomes, and evidence bases across tumor types for side-by-side comparison without implying equivalent evidence strength or conserved function.

### 4.1. Gastric Cancer

In gastric cancer (GC), UPP1 is commonly upregulated in tumor tissues and malignant epithelial populations, but its reported biological and clinical associations vary with disease state and treatment exposure [61,62,63]. Liu et al. [62] identified an ARNT–UPP1 positive-feedback loop in gastric cancer. In patient cohorts, this program was associated with adverse prognosis, whereas perturbation experiments in cell and mouse models supported its functional contribution to glycolytic remodeling and tumor growth. A complementary Helicobacter pylori-associated pattern links NF-κB/p65 activation to increased UPP1 expression across premalignant and malignant gastric epithelial states [45].

Under glucose restriction, the *H. pylori*–NF-κB/p65–UPP1 axis provides the strongest functional evidence in GC, linking UPP1 to uridine-supported energy pro-duction and growth [41]. Single-cell and spatial transcriptomics further localized UPP1 to *H. pylori*-positive, intestinal metaplasia-like, and malignant epithelial populations, with expression increasing along trajectories toward cancer-like states; patient tissues and organoid experiments linked this pattern to gastric epithelial lineage remodeling [63].

Clinical associations are less uniform. UPP1 appears in multigene prognostic models [74,75], whose risk estimates apply to the complete signatures. In a subset of a retrospective 5-FU-treated cohort, higher “UP” expression in tumor than paired non-tumor tissue was associated with a favorable outcome; the assay was reported not to amplify UPP2 [61]. GC thus has comparatively strong evidence for UPP1 upregulation, *H. pylori*-linked regulation, and metabolic or epithelial-state effects, while its independent prognostic and predictive value remains unestablished.

### 4.2. Colorectal Cancer

In colorectal cancer (CRC), UPP1 is regulator- and treatment-dependent rather than uniformly upregulated. CBFβ–RUNX2 has the broadest progression-associated support across patient tissues and in vivo models [40], whereas HOTAIR–EZH2 links UPP1 expression to uridine-supported growth through omics and in vitro perturbation [42]. The regulator-level progression evidence is broader than direct tests of UPP1 dependence.

Treatment studies reveal opposite UPP1 states in molecularly defined settings: CB-839/Nrf2-dependent induction sensitized PIK3CA-mutant CRC to 5-FU, whereas LINC01764-associated suppression reduced 5-FU activation and favored resistance [33,34]. UPP1 knockout supported the first route in xenografts; UPP1-specific validation of the second was confined to cell-based experiments.

CRC evidence is strongest for fluoropyrimidine response in selected molecular models; regulatory background and treatment exposure are more informative than tumor-wide UPP1 abundance.

### 4.3. Pancreatic Ductal Adenocarcinoma

In pancreatic ductal adenocarcinoma (PDAC), Nwosu et al. [32] combined nutrient-utilization screening across 19 human PDAC cell lines and two non-malignant pancreatic lines with UPP1 expression analysis, genetic perturbation, [^13^C_5_]uridine tracing in cultured cells and syngeneic tumors, patient-cohort analyses, and immunocompetent tumor models. This convergence gives PDAC one of the strongest cancer-specific evidence bases for UPP1-linked nutrient adaptation; the model-specific isotopologue results are detailed in Section 3.1.

Other studies broaden the PDAC pattern beyond nutrient-stressed tumor cells. Patient-tissue proteomics found greater UPP1 abundance in pancreatic cancer than in adjacent tissue and higher levels in poorly differentiated tumors [64]. Single-cell analyses of primary and metastatic disease identified UPP1-enriched cancer-associated fibroblasts in metastatic lesions, alongside exhausted CD8^+^ T-cell and complement-related macrophage programs; these parallel findings define a stromal association rather than a demonstrated immune effect of fibroblast UPP1 [46]. In a distinct intervention model, CRISPRa-engineered adipocytes with increased UPP1 competed with pancreatic cancer cells for uridine, suppressing cancer-cell growth in co-culture and reducing xenograft growth [38].

Together, these studies define three non-equivalent PDAC contexts: tumor-cell nutrient dependence in the glucose-restricted model, UPP1-enriched fibroblast states in metastatic tissue, and engineered adipocyte-mediated uridine competition.

### 4.4. Lung Adenocarcinoma

In lung adenocarcinoma (LUAD), UPP1 is enriched in invasion-associated tumor-cell niches and is associated with poorer survival and coordinated immunosuppressive cellular states [36]. Unlike association-dominant tumor settings, the same study combined patient-derived mapping with tumor-cell perturbation, pathway rescue, co-culture, and syngeneic anti-PD-L1 experiments [36]. The resulting evidence supports a tumor-cell UPP1-linked immune mechanism in LUAD, but the use of UPP1 status to guide treatment selection remains preclinical.

### 4.5. Glioma and Glioblastoma

Across glioma cohorts, UPP1 upregulation is associated with poorer survival and immune-related transcriptional states [52,53]. UPP1 silencing reduced tumor-cell proliferation and migration as well as microglial migration in vitro [53], but these experiments did not test checkpoint signaling or T-cell function.

Glioblastoma studies add checkpoint-associated and epigenetic patterns. IDH-wild-type cohorts linked UPP1 to immune infiltration, checkpoint-related features, and prognostically informative CpG sites [55]; additional studies placed UPP1 within a grade-linked methylation pattern [76], a six-gene prognostic model [65], and a c-Jun-regulated transcriptional network [77]. Together, these findings define an immune-associated and epigenetically distinct UPP1 state rather than a resolved immune mechanism.

### 4.6. Hepatocellular Carcinoma

Hepatocellular carcinoma (HCC) encompasses etiologically and molecularly diverse states, illustrated by HBV rtA181T-associated mutational pressure and PSMD12–CDK1-dependent cell-cycle progression [78,79,80]; neither line of work examines UPP1 or uridine metabolism. UPP1-focused evidence comes from two HepG2 studies: HGF-dependent induction enhanced intracellular 5-fluorouridine formation and 5-FU cytotoxicity [35], whereas CPBMF65-dependent inhibition increased intracellular uridine and induced cell-cycle arrest and senescence [43].

Uridine-centered studies define a separate HCC pattern. HCC tissues contained less uridine and showed higher CAD and DHODH expression than adjacent non-tumor tissues, while exogenous uridine suppressed HCC-cell growth and tumor growth through ferroptosis-associated effects [68]. Nrf2 protein abundance increased after ur-idine treatment, but its contribution to the pro-ferroptotic phenotype was not tested. In sepsis-associated acute lung injury, UPP1 was identified in transcriptomic and tissue expression analyses, whereas the functional experiments again manipulated uridine rather than UPP1. In macrophage models, uridine activated Nrf2 signaling and suppressed ferroptosis [81]. The opposite ferroptotic directions are consistent with disease- and cell-state dependence of uridine effects, but they do not establish opposing functions of UPP1.

Thus, the HCC literature contains direct cell-based UPP1 evidence for fluoropyrimidine response and intracellular uridine homeostasis, alongside separate uridine-centered evidence for ferroptosis. UPP1 perturbation was not tested in the HCC ferroptosis study.

### 4.7. Other Cancer Contexts

Evidence in prostate adenocarcinoma (PRAD) and cervical or other gynecologic cancers is predominantly subtype- or signature-based. In PRAD, UPP1 was nominated as a candidate antigen within immune subtypes differing in prognosis and antigen-presenting-cell infiltration [73]. In cervical cancer, UPP1 formed part of a six-gene immune-related signature associated with survival, tumor-microenvironmental features, and predicted immune-checkpoint-inhibitor response [66]. A separate circadian-transcriptomic analysis included UPP1 in a six-gene cervical-cancer prognostic model within a broader gynecologic comparison [67]. These estimates apply to the subtype or multigene signature rather than to UPP1 alone.

Oral squamous cell carcinoma (OSCC) provides the clearest UPP1-specific evidence in this subsection. Paired patient tissues and functional models linked reduced KMT5C activity to increased UPP1 expression. NR2C2-recruited KMT5C increased H4K20me3 at the UPP1 promoter and repressed UPP1 transcription; KMT5C perturbation altered tumor growth and metastasis in vitro and in vivo, and UPP1 inhibition partly reversed the effects of KMT5C loss [69].

Patient-derived, in vitro, and in vivo models link UPP1 upregulation to AKT activation, FOXO1/DCK suppression, and gemcitabine resistance in bladder cancer [37], defining a treatment-specific resistance mechanism.

Esophageal squamous cell carcinoma (ESCC) illustrates cell-source and spatial-metabolic evidence. Single-cell transcriptomics identified UPP1 among metabolism-related genes in endothelial compartments embedded within angiogenic and motility-associated programs, without direct UPP1 perturbation [70]. Spatial metabolomics separately found a higher uracil-to-uridine ratio and stronger “UPase1” staining in cancer regions [71]. This concordance is compatible with increased local uridine phosphorolysis but does not quantify UPP1-specific flux or establish causality.

In clear cell renal cell carcinoma (ccRCC), MAZ activated both TYMP and UPP1, and the authors proposed a co-regulated TYMP–UPP1 nucleotide-metabolism complex [72]. Promoter hypomethylation, clinicopathological stage, and survival associations were demonstrated primarily for TYMP, whereas UPP1 was supported by MAZ-dependent co-regulation and interaction data. Among these contexts, OSCC has the clearest UPP1-specific epigenetic and functional chain; PRAD and cervical cancer are signature-based, ESCC is spatial and endothelial, and ccRCC supports MAZ-dependent co-regulation rather than an independent UPP1 biomarker or dependency.

## 5. Clinical Implications

UPP1 has not entered routine clinical practice as an independent prognostic or predictive biomarker, and no UPP1-directed therapy has been clinically established. The current evidence consists of retrospective tissue and cohort analyses, multigene risk models, preclinical intervention studies, and one exploratory phase I evaluation in a fluoropyrimidine-combination setting [33]. Its present clinical relevance falls into four distinct roles: prognostic association, treatment prediction, pharmacodynamic or pathway-engagement assessment, and therapeutic modulation.

Prognostic evidence varies in assay design and degree of UPP1 specificity. In oral squamous cell carcinoma, historical UPase immunoreactivity at the invasive front was associated with lymph-node metastasis and shorter overall survival, although cross-reactivity with the subsequently identified UPP2 isoenzyme was not directly assessed [82]. In thyroid cancer, higher UPP1 expression correlated with adverse clinicopathological features, and UPP1 loss reduced migration, invasion, and proliferation in vitro [83]. In glioblastoma, UPP1 contributed to a methylation-based multigene risk model rather than functioning as a stand-alone marker [65]. The treated gastric-cancer cohort reported this favorable association in a subset of patients, but its design could not separate prognosis from treatment prediction [61].

Predictive and pharmacodynamic evidence is most developed in fluoropyrimidine treated settings. In PIK3CA-mutant colorectal cancer, CB-839-induced UPP1 increased 5-FU activation in preclinical models; the accompanying phase I study yielded an exploratory signal [33]. The broader LINC01764–hnRNPK–c-MYC program was linked to poor chemotherapy response across patient-cohort, cell-based, and mouse analyses, whereas the UPP1-specific reduction in 5-FU activation was demonstrated in vitro [34]. Intracellular 5-fluorouridine formation [35] and fluoropyrimidine-metabolite or nucleotide-pool changes [33] demonstrate pathway engagement experimentally, but these readouts have not been validated as clinical pharmacodynamic assays. Clinical prediction would need to integrate UPP1 with genotype, metabolic state, and treatment exposure.

Therapeutic implications are direction-dependent. Maintaining or inducing tumor-cell UPP1 enhanced fluoropyrimidine activation in selected models [33,35]; suppressing tumor-cell UPP1 reduced nutrient-dependent growth or gemcitabine resistance [32,37]. Direct UPP1 inhibition also reduced HepG2 proliferation in vitro [43]. Outside tumor cells, inhibition limited uracil-associated niche remodeling in a metastasis model [17], while UPP1-engineered adipocytes deprived pancreatic cancer cells of uridine [38]. Each strategy acts through a different cellular source and biological output, and none has established clinical efficacy.

At present, clinical interpretation of UPP1 requires five qualifiers: isoform specificity, cellular source, molecular background, demonstrated biochemical or signaling activity, and treatment context. Retrospective associations, multigene signatures, metabolite measurements, and treatment-response models contribute different information. UPP1 is best positioned as one component of an integrated molecular and metabolic profile rather than as a stand-alone pan-cancer marker [84,85].

## 6. Limitations of Current Evidence

The UPP1 literature is weighted toward transcriptomic, proteomic, single-cell, and retrospective analyses [26,46,52,53,54,55,64]. Functional intervention studies are concentrated in PDAC nutrient adaptation, LUAD immune remodeling, selected fluoropyrimidine models, HepG2 inhibition, and bladder-cancer gemcitabine resistance [32,33,34,35,36,37,43]. UPP1-specific in vivo validation is available in PDAC nutrient adaptation, LUAD immune remodeling, the CB-839/5-FU model, and bladder-cancer gemcitabine resistance [32,33,36,37]. Prospective clinical validation of UPP1 as an independent biomarker or therapeutic target is absent. Expression-based observations therefore identify candidate settings for perturbation rather than demonstrate functional dependence [86].

Metabolic measurements resolve different biological quantities. Expression and protein abundance do not measure enzyme activity, while steady-state metabolite pools do not define substrate origin, pathway direction, or reaction rate. Uptake or product-formation assays measure substrate use or product generation [17,35,38]; extracellular acidification rate (ECAR) and oxygen consumption rate (OCR) provide global bioenergetic rate readouts [39,41]; isotopologue distributions establish substrate fate and relative labeling [16,32]; and purified-enzyme kinetics define catalytic properties [20]. None of these measurements alone yields model-derived absolute pathway flux [48,49]. Spatial metabolomics adds regional information but not isoform-specific causality: in ESCC, concordant uridine/uracil changes and “UPase1” staining localized a cancer-region-associated pattern without measuring UPP1-specific flux [71]. Each assay therefore supports a distinct level of inference [48,49,87].

Cellular and spatial attribution is incomplete. Bulk tissue measurements combine tumor, immune, and stromal compartments and may assign niche-restricted activity to the wrong cellular source. In a metastasis model, neutrophils were a major UPP1 source within the pre-metastatic lung niche [17], while other studies identified UPP1-enriched fibroblasts or experimentally modified adipocytes [38,46]. Most studies did not resolve local uridine or uracil availability, leaving niche-level turnover unresolved.

UPP1 specificity is also complicated by related enzymes and incompletely defined regulation. UPP2 and TYMP are plausible sources of compensation for phenotypes driven by substrate turnover or treatment-related metabolite formation, but this has not been demonstrated [23,24,28]. In immune and stromal settings, cell-selective perturbation, metabolite measurement, and immune-function readouts are rarely combined within the same model [17,36,38,46,52,53,54,55]. No study reviewed here functionally tested any post-translational modification of UPP1; candidate-site annotations have not been linked to protein stability, localization, enzyme activity, uridine turnover, or phenotype [88].

Clinical interpretation is constrained by retrospective cohorts, heterogeneous assays, multigene signatures, and historical UP/UPase nomenclature. Treated-cohort associations do not by themselves separate prognostic from predictive value [61], and multigene models cannot assign performance to UPP1 alone [65,74,75]. Historical as-says require case-by-case scrutiny: the gastric-cancer primers were reported not to hybridize to UPP2 [61], whereas cross-reactivity of the historical OSCC UPase antibody was not directly assessed [82]. Prospective clinical designs therefore need isoform-specific, pre-specified assays and explicit documentation of treatment exposure and assay design [84,85].

Across the reviewed literature, the principal unresolved distinction is between context-specific function and non-redundant metabolic dependency. Several models establish the former through matched perturbation and pathway-proximal measurements, but few metabolic studies test compensatory enzymes sufficiently to establish the latter.

## 7. Conclusions

UPP1 expression alone is insufficient to establish biological relevance or predict phenotypic direction. Functional relevance is best supported when UPP1 perturbation, a pathway-proximal readout, and a phenotype measured in the same defined setting converge. The resulting effect depends on the process chiefly engaged, including nutrient-stress carbon utilization, fluoropyrimidine activation, AKT–DCK-related gemcitabine resistance, tumor-cell cytokine and immune-checkpoint signaling, and cell-source-specific redistribution of uridine or uracil. Models that align perturbation with pathway-proximal measurement and an in vivo phenotype currently provide the strongest evidence. Accordingly, UPP1 should be interpreted through pathway activity, cellular source, substrate state, molecular background, treatment exposure, and potential compensation rather than expression level alone.

## 8. Future Directions

The first priority is to determine whether UPP1 is a non-redundant metabolic requirement in a defined tumor state. Mapping UPP1, UPP2, and TYMP expression and enzymatic activity in the relevant cell population should precede matched genetic and pharmaco-logical perturbations, including single and paired interventions coupled to metabolite rescue, uridine/uracil turnover, and phenotype measurements [23,24,28,88,89]. Stable-isotope tracing can then resolve uridine-derived carbon fate; quantitative flux analysis is appropriate only when temporal and mass-balance constraints are included [16,32,48,49]. For fluoropyrimidine models, drug-metabolite readouts should accompany these analyses [33,34,35]. Gemcitabine models instead require target-proximal AKT–FOXO1–DCK readouts to test a UPP1-specific signaling requirement [37]. Together, these designs would distinguish non-redundant metabolic dependence from UPP1-specific signaling functions and identify the settings in which each is functionally required.

A second priority is to resolve cellular source and spatial organization within the same experimental unit. Cell-type-resolved expression and selective perturbation should be integrated with targeted uridine/uracil metabolomics, isotope-resolved analysis, and spatial metabolomics so that UPP1 abundance, local substrate–product patterns, and immune or stromal phenotypes are linked within the same tissue region [17,38,46,48,49,71,87,90,91]. Architecture-preserving tissue technologies can additionally assign these measurements to defined cellular neighborhoods [92]. Linking the measurements within the same region would distinguish tumor-cell dependence from metabolite redistribution driven by neutrophils, fibroblasts, adipocytes, or other non-malignant compartments.

Redox and ferroptosis provide a distinct test case for cell-state-dependent UPP1 function. The opposing uridine responses reported in HCC and sepsis offer a paired system for testing whether UPP1-mediated turnover influences ferroptosis sensitivity [68,81]. Parallel tumor-cell and myeloid-cell models should combine UPP1 loss- and gain-of-function with controlled uridine supplementation, measurement of intracellular and extracellular uridine, Nrf2 perturbation, and ferroptosis-specific rescue and readouts. Ferroptosis-focused glioblastoma work provides a methodological precedent for species-resolved lipidomics and oxidized-phospholipid profiling [93]. The paired design would test whether the direction of ferroptotic response is set by cellular identity, disease state, or their interaction.

Regulatory studies need to connect UPP1 expression control to enzyme activity and pathway dependence. Pan-cancer datasets can prioritize tumor states for mechanistic investigation [94], while the transcriptional and epigenetic programs implicated in gastric and colorectal cancer provide defined backgrounds in which to test enzyme activity, uridine turnover, and phenotype rescue [40,41,42,45]. Candidate post-translational regulation should first be established at the UPP1 protein level and then tested by site-directed mutagenesis together with measurements of protein stability, subcellular localization, enzyme activity, uridine turnover, and phenotype. Paired activity and rescue measurements would distinguish regulatory features that accompany UPP1 abundance from those that alter catalytic function or cellular dependency.

Translational programs should begin in mechanistically justified tumor states and predefine the intended direction of UPP1 modulation. Each program should determine whether inhibition, maintenance, induction, or cell-source-specific redistribution is appropriate; establish an isoform-specific assay and an on-target pathway-engagement marker; and assess normal-tissue effects and the therapeutic window, given the biological activity of UPP1 inhibition in non-malignant liver-fibrosis models [95]. Assay plans should account for reproducibility and spatial heterogeneity, drawing on protein biomarker studies [96], architecture-preserving tissue platforms [92], comprehensive genomic profiling [97], and metabolite-centered measurements [98]. Molecular and metabolic eligibility should then be pre-specified and tested prospectively before a UPP1-directed treatment-development study [84,85].

## Figures and Tables

**Table 1:** Representative Mechanistic Axes Linking UPP1 to Metabolic Adaptation, Immune Remodeling, and Treatment-Related Response.

Mechanistic Context	Regulator/Input	UPP1-Linked Process	Reported Outcome	Evidence Basis	Ref.
Nutrient stress	Uridine/RNA	R1P generation and ribose-carbon salvage	Glycolytic support	In vitro; in vivo stable-isotope tracing ^1^	[16]
LUAD glycolysis	Histone acetylation	Glucose uptake; lactate production	Proliferation; 2-DG sensitivity	Omics; clinical; in vitro; in vivo	[39]
CRC transcription	CBFβ–RUNX2	UPP1 promoter activation	Progression-associated program involving growth and metastasis	Clinical; in vitro; in vivo	[40]
GC inflammation	*H. pylori*–NF-κB/p65	UPP1 transcription; uridine utilization	Glycolytic adaptation; growth	Omics; in vitro; organoid; clinical	[41]
CRC epigenetics	HOTAIR–EZH2	H3K27me3-associated UPP1 regulation	Uridine-supported growth	Omics; in vitro	[42]
PDAC nutrient adaptation	KRAS–MAPK; glucose restriction	Uridine-ribose utilization	Redox support; survival; growth	Clinical; in vitro; in vivo; stable-isotope tracing	[32]
LUAD immune remodeling	UPP1 upregulation	TGF-β1; PI3K–AKT–mTOR; PD-L1	Treg enrichment; CD8^+^ T-cell dysfunction; preclinical anti-PD-L1 response	Omics; clinical; in vitro; in vivo	[36]
CRC 5-FU sensitization	CB-839–ROS–Nrf2	UPP1 induction; 5-FU activation	5-FU sensitization	Omics; in vitro; in vivo; clinical ^2^	[33]
CRC 5-FU resistance	LINC01764–hnRNPK–c-MYC	UPP1 suppression	5-FU resistance	Omics; clinical; in vitro; in vivo ^3^	[34]
HepG2 inhibition	CPBMF65	UPP1 inhibition; uridine accumulation	Cell-cycle arrest; senescence	In vitro	[43]
HepG2 5-FU response	HGF–c-Met	UPP1 induction; fluorouridine formation	5-FU sensitization	In vitro	[35]
BLCA drug resistance	UPP1–AKT	AKT activation; FOXO1/DCK suppression	Growth; metastasis; gemcitabine resistance	Omics; clinical; in vitro; in vivo	[37]

Evidence categories are non-mutually exclusive. Clinical evidence includes patient-derived, retro-spective, or exploratory clinical data and does not imply prospective validation. Stable-isotope tracing denotes labeled-substrate fate or isotopologue enrichment and does not imply model-derived absolute flux unless quantitative flux analysis was explicitly reported. ^1^ Non-tumor in vivo stable-isotope tracing. ^2^ Exploratory phase I evidence. ^3^ Direct UPP1 validation of 5-FU resistance was primarily in vitro. Abbreviations: 2-DG, 2-deoxy-D-glucose; 5-FU, 5-fluorouracil; AKT, protein kinase B; BLCA, bladder cancer; CRC, colorectal cancer; DCK, deoxycytidine kinase; GC, gastric cancer; HGF, hepatocyte growth factor; HOTAIR, HOX transcript antisense RNA; LUAD, lung adenocarcinoma; NF-κB, nuclear factor kappa B; Nrf2, nuclear factor erythroid 2-related factor 2; PD-L1, programmed death-ligand 1; PDAC, pancreatic ductal adenocarcinoma; Treg, regulatory T cell.

**Table 2:** Cancer-Type-Specific Patterns of UPP1 Across Tumor Contexts.

Tumor	UPP1 Pattern	UPP1-Associated Context	Reported Outcome	Evidence Basis	Ref.
CRC	Regulator- and treatment-dependent	CBFβ–RUNX2; HOTAIR–EZH2; LINC01764–c-MYC; ROS–Nrf2	Progression; metastasis; 5-FU response	Omics; clinical; in vitro; in vivo	[33,34,40,42]
GC	Upregulated	*H. pylori*–NF-κB/p65; ARNT; IM enrichment	Glycolytic adaptation; progression; prognosis; 5-FU-associated outcome	Omics; clinical; in vitro; organoid; in vivo	[41,45,61,62,63]
LUAD	Niche-enriched	TGF-β1; PI3K–AKT–mTOR; PD-L1	Invasion; immunosuppression; prognosis; preclinical anti-PD-L1 response	Omics; clinical; in vitro; in vivo	[36]
PDAC	Nutrient-inducible	KRAS–MAPK; uridine-ribose utilization; stromal enrichment	Nutrient adaptation; growth; prognosis; uridine dependence	Omics; clinical; in vitro; in vivo; stable-isotope tracing	[32,38,46,64]
Glioma	Upregulated	Immune-evasion programs	Prognosis; proliferation; invasion	Omics; clinical; in vitro	[52,53]
GBM	Upregulated	Checkpoint signatures; CpG methylation	Prognosis; immune infiltration; methylation stratification; tumor-cell migration	Omics; clinical; in vitro	[55,65]
Cervical cancer	Signature-linked	Immune/circadian signatures	Prognosis; model-predicted ICI response	Omics; clinical	[66,67]
HCC	Experimentally modulated	HGF–UPP1; UPP1 inhibition; uridine–ferroptosis	5-FU sensitization; growth suppression	Clinical; in vitro; in vivo ^1^	[35,43,68]
BLCA	Upregulated	UPP1–AKT; FOXO1/DCK	Progression; metastasis; gemcitabine resistance	Omics; clinical; in vitro; in vivo	[37]
OSCC	Upregulated	NR2C2–KMT5C–H4K20me3	Proliferation; invasion; metastasis	Clinical; in vitro; in vivo	[69]
ESCC	Endothelial- and cancer-region-enriched	Endothelial program; spatial uridine/uracil pattern	Association with angiogenic and endothelial-motility programs; regional uridine/uracil alteration	Omics; spatial metabolomics; clinical	[70,71]
ccRCC	MAZ-regulated	MAZ–TYMP–UPP1; nucleotide metabolism	Methylation-linked progression ^2^	Omics; clinical; in vitro	[72]
PRAD	Subtype-linked	Immune subtype; candidate antigen	Prognostic/immune stratification	Omics; clinical	[73]

Evidence categories are non-mutually exclusive. Clinical evidence includes patient-derived, retro-spective, or exploratory clinical data and does not imply prospective validation. Stable-isotope tracing denotes labeled-substrate fate or isotopologue enrichment and does not imply mod-el-derived absolute flux unless quantitative flux analysis was explicitly reported. ^1^ In Ref. [68], the patient-derived and in vivo evidence is uridine-centered rather than UPP1-specific; the UPP1-specific evidence in Refs. [35,43] is in vitro. ^2^ In Ref. [72], clinical associations primarily con-cern TYMP methylation; UPP1 is implicated through MAZ co-regulation. Abbreviations: 5-FU, 5-fluorouracil; AKT, protein kinase B; ARNT, aryl hydrocarbon receptor nuclear translocator; BLCA, bladder cancer; CRC, colorectal cancer; DCK, deoxycytidine kinase; ESCC, esophageal squamous cell carcinoma; GC, gastric cancer; GBM, glioblastoma; HCC, hepatocellular carcinoma; HGF, hepatocyte growth factor; HOTAIR, HOX transcript antisense RNA; ICI, immune checkpoint inhibitor; IM, intestinal metaplasia; LUAD, lung adenocarcinoma; NF-κB, nuclear factor kappa B; Nrf2, nuclear factor erythroid 2-related factor 2; OSCC, oral squamous cell carcinoma; PD-L1, programmed death-ligand 1; PDAC, pancreatic ductal adenocarcinoma; PRAD, prostate adenocarcinoma; TYMP, thymidine phosphorylase; ccRCC, clear cell renal cell carcinoma.

## Data Availability

No new experimental data were generated in this study. The approximate isotopologue percentages reported in Section 3.1 for Ref. [16] were calculated by the authors from the publicly available peak-area data in Source Data Figure 2 accompanying that publication.

## Abbreviations

5-FU	5-fluorouracil
AKT	protein kinase B
ARNT	aryl hydrocarbon receptor nuclear translocator (also known as HIF-1β)
CAF	cancer-associated fibroblast
CAD	carbamoyl-phosphate synthetase 2, aspartate transcarbamylase, and dihydroorotase
CBFβ	core-binding factor subunit beta
ccRCC	clear cell renal cell carcinoma
CD8	cluster of differentiation 8
CRC	colorectal cancer
CRISPRa	CRISPR activation
DCK	deoxycytidine kinase
DHODH	dihydroorotate dehydrogenase
EGFR	epidermal growth factor receptor
ESCC	esophageal squamous cell carcinoma
EZH2	enhancer of zeste homolog 2
FOXO1	forkhead box O1
GBM	glioblastoma
GC	gastric cancer
HCC	hepatocellular carcinoma
HGF	hepatocyte growth factor
HIF-1α	hypoxia-inducible factor 1 alpha
HOTAIR	HOX transcript antisense RNA
H3K27me3	histone H3 lysine 27 trimethylation
H4K20me3	histone H4 lysine 20 trimethylation
ICI	immune checkpoint inhibitor
IDH	isocitrate dehydrogenase
KMT5C	lysine methyltransferase 5C
KRAS	Kirsten rat sarcoma viral oncogene homolog
LAG3	lymphocyte activation gene 3
LUAD	lung adenocarcinoma
MAPK	mitogen-activated protein kinase
MAZ	MYC-associated zinc-finger protein
MDSC	myeloid-derived suppressor cell
mTOR	mechanistic target of rapamycin
NF-κB	nuclear factor kappa B
Nrf2	nuclear factor erythroid 2-related factor 2
NR2C2	nuclear receptor subfamily 2 group C member 2
OSCC	oral squamous cell carcinoma
PD-1	programmed cell death protein 1
PD-L1	programmed death-ligand 1
PDAC	pancreatic ductal adenocarcinoma
PI3K	phosphoinositide 3-kinase
PIK3CA	phosphatidylinositol-4,5-bisphosphate 3-kinase catalytic subunit alpha
PPP	pentose phosphate pathway
PRAD	prostate adenocarcinoma
PTEN	phosphatase and tensin homolog
R1P	ribose-1-phosphate
ROS	reactive oxygen species
TAM	tumor-associated macrophage
TGF-β1	transforming growth factor beta 1
TME	tumor microenvironment
TP53	tumor protein p53
Treg	regulatory T cell (plural Tregs)
TYMP	thymidine phosphorylase
UCK1/UCK2	uridine-cytidine kinases 1 and 2
UP	uridine phosphorylase (historical abbreviation; isoenzyme identity depends on the assay design)
UPase	uridine phosphorylase (historical nomenclature; not automatically equivalent to UPP1)
UPase1	uridine phosphorylase 1 (terminology used in the original ESCC study)
UPP1	uridine phosphorylase 1
UPP2	uridine phosphorylase 2
